# Analysis of endometrial thickness patterns and pregnancy outcomes considering 12,991 fresh IVF cycles

**DOI:** 10.1186/s12911-021-01538-2

**Published:** 2021-06-03

**Authors:** ShuJie Liao, Renjie Wang, Cheng Hu, Wulin Pan, Wei Pan, Dongyang Yu, Lei Jin

**Affiliations:** 1 Department of Obstetrics and Gynecology, Tongji Hospital, Tongji Medical College, Huazhong University of Science and Technology, Wuhan, 430030 Hubei People’s Republic of China; 2 School of Economic and Management, Wuhan University, Wuhan, 430072 People’s Republic of China; 3 School of Applied Economics, Renmin University of China, Beijing, 100872 People’s Republic of China

**Keywords:** In vitro fertilization, Endometrial thickness, Endometrial pattern, Clinical pregnancy

## Abstract

**Background:**

Different endometrial patterns have an important effect on the relationship between endometrial thickness (EMT) and clinical pregnancy rate. There is a significant difference in age, selection of cycle protocols, and clinical pregnancy rates among four groups with diverse endometrial patterns.

**Methods:**

This retrospective study aimed to assess the association between EMT on human chorionic gonadotropin (HCG) administration day and the clinical outcome of fresh in vitro fertilization (IVF). The 5th, 50th, and 95th percentiles for EMT were determined as 8, 11, and 14 mm, respectively. Patients were sub-divided into four groups based on their EMT in different endometrial patterns (Group 1: < 8 mm; Group 2: ≥ 8 and ≤ 11 mm; Group 3: > 11 and ≤ 14 mm; Group 4: > 14 mm). We divided patients into three groups based on their endometrial pattern and evaluated the correlation between EMT and clinical pregnancy rate.

**Results:**

We found a positive correlation between pregnancy rates and EMT in all endometrial patterns. Multiple logistic regression analysis proved age, duration of infertility, cycle protocols, number of embryos transferred, progesterone on HCG day, endometrial patterns, and EMT have significant effects on clinical pregnancy rates. Meanwhile, there was a significant difference in age, selection of cycle protocols, and clinical pregnancy rates among four groups with diverse endometrial patterns.

**Conclusions:**

Different endometrial patterns have an important effect on the relationship between EMT and clinical pregnancy rate.

## Background

IVF technology has helped many people who cannot naturally get pregnant solve their infertility. Many clinical factors are used to evaluate the assisted reproductive technology (ART) outcomes, including EMT and endometrial pattern, which greatly influence pregnancy outcomes [15, 19].

Some researchers have examined the relationship between EMT and pregnancy complications [13, 14, 22]. Previous research indicates that EMT and clinical pregnancy rate were positively correlated [2, 8, 10, 16, 18]. Holden et al. concluded that EMT < 6 mm had adverse effects on clinical pregnancy and live birth rates following initial IVF cycles [7]. A meta-analysis by Gao et al. explored whether EMT could predict pregnancy outcomes after IVF. They suggested that lower EMT was associated with a lower incidence of pregnancy rate, implantation rate, and live birth or ongoing pregnancy rate [5]. Studies suggest that EMT has little effect on pregnancy outcomes. Bu et al. found that a thick endometrium did not have a detrimental effect on IVF outcome [4].

Zhao et al. [21] stated that EMT and endometrial pattern had no predictive value on the outcome of in vitro fertilization-embryo transfer (IVF-ET) [20]. Weiss et al. suggested that differences in EMT between pregnant women and non-pregnant women were too small to guide individual treatments [17]. However, other studies had opposite conclusions. Researchers found that a homogenous, hypoallergenic endometrium had a detrimental effect on clinical pregnancy rate [6]. Kasius et al. [9] found it hard to identify women unlikely to conceive after IVF based on EMT. A significant relationship between EMT and IVF results was not apparent, and their value remains controversial.

Our study combined EMT and endometrial patterns to analyze their impact on clinical pregnancy rates. Moreover, most of the former research samples primarily included Caucasian subjects, while we focus on Asian individuals. We also have an important sample size and believe our research can further understanding of the relationship between EMT and pregnancy rates. Our patients were divided into three groups based on their endometrial pattern to accurately evaluate the correlation between EMT and clinical pregnancy rate.

## Methods

### Patient database

A retrospective cohort of 12,991 in vitro fertilization-intracytoplasmic sperm injection (IVF-ICSI) cycles was enrolled at the Reproductive Medicine Center of Tongji Hospital, PR China from 2014 to 2017. We included fresh IVF–ICSI cycles and embryo transfers within the study period, regardless of the infertility diagnosis, reproductive history, body mass index, or insemination method. Cycles using donor oocytes or suspect endometrial abnormalities were excluded. The cause of infertility was categorized as ovary-related factors, tubal-related factors, endometriosis, male factors, uterine factors, other (including unexplained factors), and multiple factors. The Data Analysis Center of Tongji Hospital performed all data acquisition, management, and analyses.

The patients provided personal information such as age and duration of infertility. Professional medical technicians obtained other factors, including estradiol (E2) and progesterone levels on day 1 of stimulation, follicle-stimulating hormone (FSH), and the number of oocytes retrieved.

All transvaginal ultrasound (TVU) assessments were performed in our center by specialist clinicians using the same standardized protocol on the same ultrasound instrument (ALOKA ColorSound ProSound SSD-3500SV, Hitachi Aloka Medical, USA). We measured EMT and endometrial patterns on the medial sagittal plane of the uterus on the day of HCG administration and the maximum thickness from one interface to the other at the junction of the endometrial muscle. Other factors measured include body mass index, cycle protocols, the number of oocytes retrieved, the number of embryos transferred, time of gonadotropins used, the number of mature follicles, HMG dose, and clinical pregnancy rate.

**Fig. 1:**
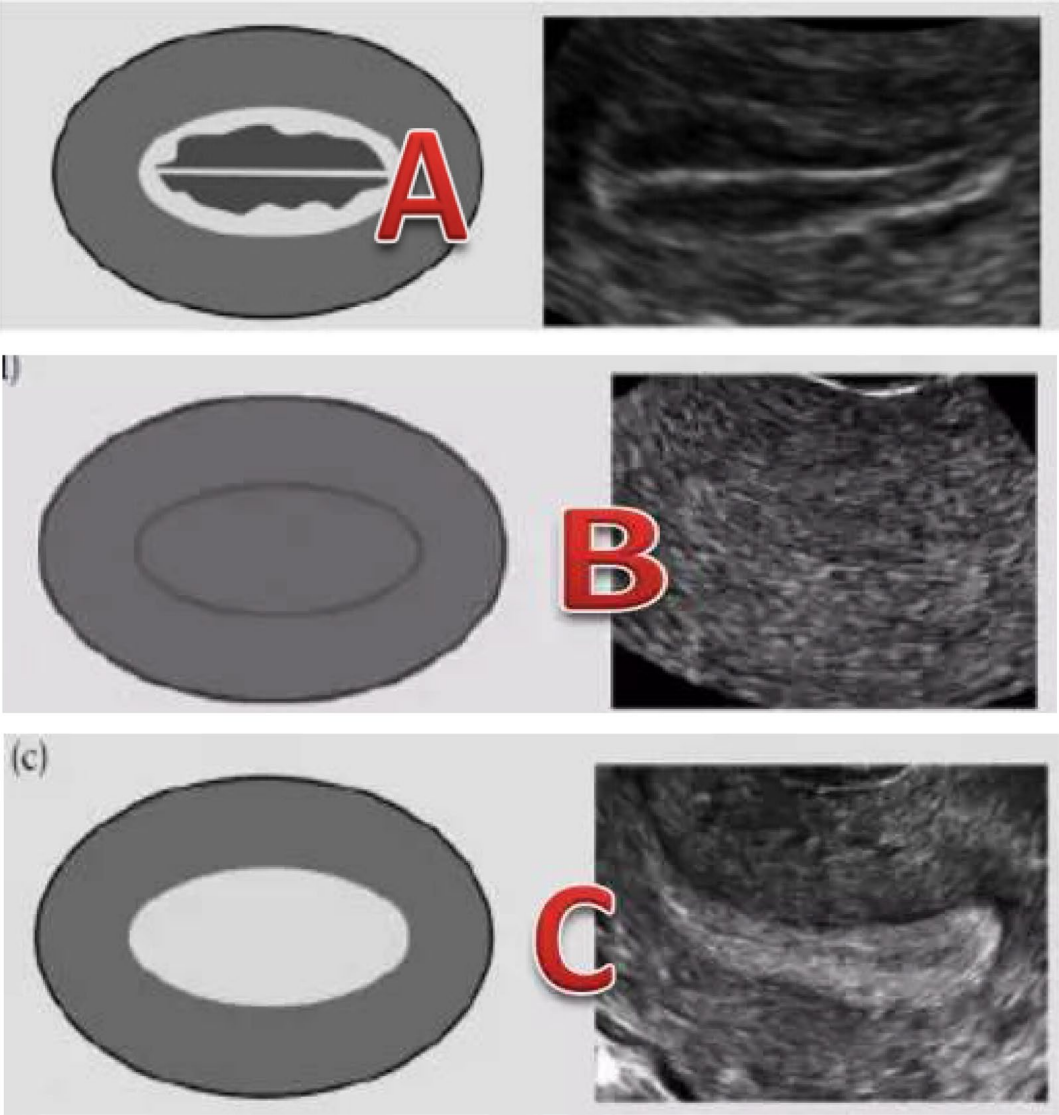
Schematic diagram of three types of endometrium

Patients were divided into four groups by EMT: Group 1 (≤ 8 mm); Group 2 (8–11 mm); Group 3 (11–14 mm); Group 4 (≥ 14 mm). Endometrial patterns were classified as type A (a triple line pattern consisting of a central hyperechoic line surrounded by two hypoechoic layers); type B (an intermediate isoechogenic pattern with the same reflectivity as the surrounding myometrium and a poorly defined central echogenic line); and type C (homogenous, hypoallergenic endometrium (Zhao et al. 2014). Images of the three endometrial patterns are shown in Fig. 1.

Ovarian stimulation protocols, including the long agonist protocol, short agonist protocol, and prolonged agonist protocol, were carried out as previously described [11, 12],Ai et al. [1]. The long agonist protocol (GnRH agonist protocol) was as follows: 0.1 mg/d Triptorelin (Diphereline, IPSEN, Paris, France) was subcutaneously injected from the midluteal phase of the last menstrual cycle. Nondiameter follicular cysts > 10 mm were defined as the reduction criterion. The patients were administered recombination follicle-stimulating hormone at 75–150 IU/day (rFSH, Gonal-F, Merck Serono, Switzerland) while Triptorelin was reduced to 0.05 mg/d.

The short antagonist protocol (GnRH antagonist protocol) was as follows: rFSH stimulation was initiated on day two or three of the menstrual cycle until ovulation induction. During the stimulation, the rFSH dosage ranged from 150 to − 225 IU, and was adjusted according to the ovarian response as evaluated by TVU. When the leading follicles reached a mean diameter of 14 mm, Cetrotide (Cetrotide, Merck Serono, Germany) was subcutaneously injected at 0.25 mg/d until ovulation induction.

The prolonged agonist protocol (GnRH agonist ultra-long protocol) was as follows: a single dose of 3.75 mg Triptorelin was administrated on the first or second day of the menstrual cycle. rFSH was initiated daily 28 days later when the endometrial thickness was < 5 mm, the follicular diameter was < 8 mm, E2 was < 50 pg/ml, and LH was < 3 IU/L, until ovulation induction. The gonadotropin dose ranged between 150 and 225 IU and was adjusted based on the ovarian response.

For all three protocols, 0.25 mg of recombinant human chorionic gonadotropin (rhCG) (Ovidrel, Merck Serono, Switzerland) was administered when at least two follicles reached a mean diameter of 18 mm. Oocytes were retrieved 36 h after rhCG injection by transvaginal ultrasound-guided single-lumen needle aspiration. ICSI was conducted according to sperm quality, considering severe male factor infertility or previous fertilization failures. Luteal phase support was initiated on the first day after oocyte retrieval. Embryo transfer was performed on day two or three after oocyte retrieval. Embryo scoring was performed according to the Istanbul consensus (Alpha Scientists in Reproductive Medicine and Eshre Special Interest Group Embryology, [3].

### Data statement

As our data belongs to Tongji Hospital, its availability is restricted and we are unauthorized to disclose it. All data acquisition and data management were performed by the Data Analysis Center of Tongji Hospital. The study was approved by the ethics committees at the Reproductive Medicine Center of Tongji Hospital, and informed consent was signed before patient participation. According to the IRB of Tongji Hospital, our study did not require an ethics review because all women participating in the study received routine IVF treatment in the hospital and no additional intervention or sampling was performed [11, 12].

### Main supporting software

R language was used for all statistical analyses. Continuous variables were analyzed by Student’s *t*-test when categorical variables were checked by the χ^2^ test. One-way ANOVA and χ^2^ tests were used to verify the differences between the four groups. We used a multivariate logistic regression model to assess the prognostic value of the various variables on clinical pregnancy. *P* < 0.05 was considered to be significant.

### Multivariate logistic regression analysis to determine the influence of different factors on pregnancy outcomes

References and univariate logistic regression usually include age grouping, causes of infertility, EMT, endometrial patterns, FSH, large follicle numbers, treatment plan, number of embryo transfers, number of eggs obtained, and flavonoids as independent variables. Multivariate unconditional regression analysis was performed on whether pregnancy was a dependent variable. Age grouping, causes of infertility, number of years of infertility, basic antral follicle groupings, number of cycles, and pregnancy grouping were selected as the multi-classification covariates (using the definition of variables to analyze the forward method based on partial maximum likelihood estimation). The final indicators included in the equation are shown in Table 1.

**Table 1:** Multivariate unconditional logistic regression analysis

Factors	Factors grouping	B	S.E,	Wals	df	Sig	Exp (B)
Age	< 25			83.775	4.000	0.000	
	25–30	0.063	0.106	0.358	1.000	0.550	1.065
	30–35	0.034	0.107	0.099	1.000	0.753	1.034
	35–40	− 0.183	0.118	2.386	1.000	0.122	0.833
	> 40	− 1.375	0.186	54.716	1.000	0.000	0.253
DOI	Less than 1 year			19.995	5.000	0.001	
	1–3 year	− 0.081	0.132	0.380	1.000	0.538	0.922
	3–6 year	− 0.104	0.131	0.631	1.000	0.427	0.901
	6–9 year	− 0.242	0.143	2.882	1.000	0.090	0.785
	9–12 year	− 0.422	0.167	6.363	1.000	0.012	0.656
	12and above	− 0.606	0.205	8.758	1.000	0.003	0.546
EMT	Group1			98.562	3.000	0.000	
	Group2	0.610	0.103	34.719	1.000	0.000	1.840
	Group3	0.806	0.104	60.242	1.000	0.000	2.239
	Group4	1.105	0.119	86.471	1.000	0.000	3.021
Endometrial pattern	A			12.339	2.000	0.002	
	B	− 0.229	0.068	11.199	1.000	0.001	0.795
	C	− 0.135	0.088	2.319	1.000	0.128	0.874
Mature follicle number	< 5			10.745	3.000	0.013	
	5–9	0.219	0.096	5.214	1.000	0.022	1.245
	10–14	0.326	0.102	10.236	1.000	0.001	1.385
	≥ 15	0.271	0.117	5.365	1.000	0.021	1.312
clinical protocol	Long			47.341	3.000	0.000	
	Short	− 0.232	0.069	11.325	1.000	0.001	0.793
	Prolong	0.411	0.079	26.875	1.000	0.000	1.508
	Other	− 0.101	0.102	0.989	1.000	0.320	0.904
	P	− 0.489	0.078	39.090	1.000	0.000	0.613
Number of embryo transfer	0 (frozen)			194.106	3.000	0.000	
	1	20.154	511.654	0.002	1.000	0.969	565,920,737.626
	2	21.041	511.654	0.002	1.000	0.967	1,373,929,027.313
	3	20.499	511.654	0.002	1.000	0.968	799,202,249.339
	constant	− 21.098	511.654	0.002	1.000	0.967	0.000

### Prediction model

**Fig. 2:**
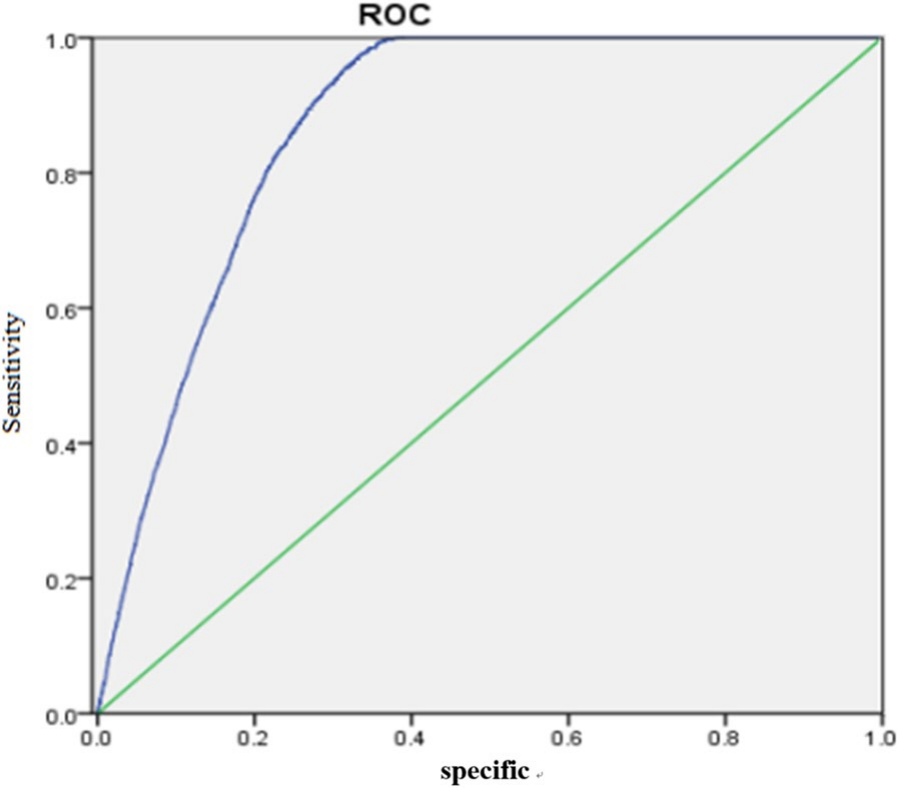
ROC curve. *Note*: The predicted value of the successful pregnancy rate is the test variable. Whether the successful pregnancy is the state variable, the sensitivity is the ordinate, and the 1-specificity is the ROC curve of the abscissa. The area under the ROC curve is equal to 0.870

The test variable was the predicted successful pregnancy rate obtained by the model. Whether successful pregnancy is the state variable or not, the sensitivity is ordinate and the 1-specificity is the ROC curve of the abscissa (Fig. 2).

The area under the ROC curve is 0.870, the standard error is 0.003, and the 95% confidence interval is ± 0.006. SPSS can be used to obtain the sensitivity and specificity pairs corresponding to different diagnostic thresholds. The graph’s coordinate points can be drawn. When *P* = 0.2452, the Yoden index is 0.640, which is considered the diagnostic boundary. The discriminating situation is shown in Table 2.

**Table 2:** Diagnostic test table

Predicting IVF results	pregnant	Not pregnant	Total
Successful pregnancy	3562 (TP)	3123 (FP)	6685
Not pregnant	94 (FN)	6212 (TN)	6306
Total	3656	9335	12,991

TP actually successful pregnancy and identify the period of successful pregnancy

FN actually successful pregnancy but discriminates the period of unsuccessful pregnancy

FP actually unsuccessful pregnancy but discriminates the cycle of successful pregnancy

TN actually unsuccessful pregnancy and discriminates the period of unsuccessful pregnancy

N = TP + FN + FP + TN

Sensitivity: TP/TP + FN = 97.42%

Specificity: TN/FP + TN = 66.54%

Correct rate = TP + TN/N = 75.23%

## Results

A total of 12,991 IVF cycles were included in our study and the clinical pregnancy rate was 28.1% (3656). Demographic data are presented in Table 3. Continuous variables are presented as means and standard deviations and categorical variables are presented as a frequency. The subjects in the pregnancy group were younger, usually chose long agonist protocols, and used lower doses of HMG, E2, and progesterone. Moreover, they had shorter periods of infertility, more embryos transferred, fewer mature follicles, a thicker endometrium, shorter gonadotropin (Gn) time, and were more type-A than that subjects in the non-pregnancy group (all variables showed significant statistical differences between the two groups). Both groups had a similar BMI and number of oocytes retrieved (no statistically significant differences). Table 4 shows the primary infertility diagnosis for subjects in both groups.

**Table 3:** demographic data of potential predictors in pregnant and non-pregnant women

Variables	Pregnancy ( +)	Non-pregnancy ( −)	*P* value
	(N = 3656)	(N = 9335)	
Age (years)	30.18 ± 4.13	32.42 ± 5.75	< 0.01
BMI (kg/m^2^)	21.88 ± 2.93	21.93 ± 2.90	0.3278
Cycle protocols			< 0.01
Long agonist protocol	2302 (63.0%)	4423 (47.4%)	
Short agonist protocol	575 (15.7%)	1870 (20.0%)	
Prolonged agonist protocol	565 (15.5%)	857 (9.2%)	
Other	214 (5.9%)	2185 (23.4%)	
Duration of infertility (years)	3.67 ± 2.68	4.25 ± 3.61	< 0.01
FSH (mIU/ml)	7.38 ± 2.57	8.05 ± 3.65	< 0.01
embryo transferred	1.86 ± 0.34	0.66 ± 0.87	< 0.01
HMG	8.05 ± 7.54	9.74 ± 8.92	< 0.01
Gntime	10 ± 1.78	9.82 ± 2.05	< 0.01
mature follicles	9.97 ± 3.93	9.47 ± 5.67	< 0.01
endometrial thickness on HCGday	11.68 ± 2.38	10.54 ± 2.81	< 0.01
E2	3362.93 ± 1756.74	3477.31 ± 2652.94	0.004228
Progesterone (ng/ml)	0.9 ± 0.30	1.23 ± 1.72	< 0.01
oocytes retrieved (n)	11.31 ± 4.59	11.33 ± 7.94	0.8667
endometrial morphology			< 0.01
a	2801 (76.6%)	5754 (61.6%)	
b	539 (14.7%)	1895 (20.3%)	
c	316 (8.6%)	1686 (18.1%)	

Data are mean ± SD or number (percentage)

*BMI* body mass index (kg/m^2^), *FSH* follicle-stimulating hormone

^a^Two-sample t-test

^b^Pearson χ^2^ test

**Table 4:** Diagnostic categories

Variables	Pregnancy ( +)	Non-pregnancy ( −)
	(N = 3656)	(N = 9335)
Ovary-related factors	114 (3.1%)	481 (5.2%)
Tubal related factors	756 (20.7%)	1599 (17.1%)
Endometriosis	42 (1.1%)	88 (0.9%)
Male factors	343 (9.4%)	705 (7.6%)
Uterine factors	37 (1%)	81 (0.8%)
Other	1344 (36.8%)	3408 (36.5%)
Multiple causes	1020 (27.9%)	2973 (31.8%)

### Statistics of patients with different endometrial patterns

Table 5 summarizes statistics for patients with triple line patterns (N = 8555), which was significantly different from the logistic regression analyses. Patients in the four different EMT groups had similar progesterone levels (*P* > 0.0.5). There was a negative association between age and EMT (34.42 ± 6.02 vs. 31.32 ± 5.00 vs. 30.4 ± 4.53 vs. 30.09 ± 4.34; *P* < 0.001). However, the proportion of patients with thinner EMTs Group 1 (< 8 mm) who chose long agonist protocols was significantly lower (28.5% vs. 54.0% vs. 59% vs. 60.1%; *P* < 0.001; 8.2% vs. 10.1% vs. 16.4% vs. 20.9%; *P* < 0.001).

**Table 5:** Predictors and clinical outcome according to EMT with tripleline patterns

Variables	Group1	Group2	Group3	Group4	*P* value
	(N = 785)	(N = 3476)	(N = 3345)	(N = 949)	
Age (years)	34.42 ± 6.02	31.32 ± 5.00	30.4 ± 4.53	30.09 ± 4.34	< 0.01
Duration of infertility (years)	4.35 ± 4.05	3.91 ± 3.16	3.95 ± 3.01	3.81 ± 2.79	0.00159
Clinical protocol					< 0.01
Long agonist protocol	224 (28.5%)	1877 (54.0%)	1973 (59%)	570 (60.1%)	
Short agonist protocol	140 (17.8%)	837(24.1%)	589 (17.6%)	121 (12.8%)	
Prolonged agonist protocol	64 (8.2%)	352 (10.1%)	548 (16.4%)	198 (20.9%)	
Other	357 (45.5%)	410 (11.8%)	235 (7.0%)	60 (6.3%)	
Embryo transferred	0.65 ± 0.88	1.13 ± 0.92	1.21 ± 0.90	1.18 ± 0.91	< 0.01
Progesterone(ng/ml)	1.08 ± 2.34	1.05 ± 0.63	1.03 ± 0.48	1.01 ± 0.41	0.261
Clinical pregnancy	96 (12.2%)	1071 (30.8%)	1250 (37.4%)	384 (40.5%)	< 0.01

^a^One-way ANOVA. Values are mean + SD

^b^Pearson χ^2^ test. Values are number (percentage)

Patients with intermediate isoechogenic patterns (N = 2434) had similar progesterone levels (*P* > 0.05) (Table 6). But the number of embryos transferred was significantly lower in patients with a thinner EMT (0.53 vs. 0.87 vs. 1.05 vs.1.07; *P* < 0.001). Patients in Group 4 were slightly older than those in Group 3 (30.9 vs. 30.8). Clinical pregnancy rates were significantly higher in patients with thicker EMTs (7.5% vs. 18.6% vs. 27.8% vs. 35.2%; *P* < 0.001).

**Table 6:** Predictors and clinical outcome according to EMT with intermediate isoechogenic pattern

Variables	Group1	Group2	Group3	Group4	*P* value
	(N = 427)	(N = 864)	(N = 769)	(N = 374)	
Age (years)	35.86 ± 5.79	32.54 ± 5.47	30.85 ± 5.13	30.93 ± 4.66	< 0.01
Duration of infertility (years)	5.17 ± 4.70	4.37 ± 3.50	4.41 ± 3.58	4.23 ± 3.35	0.000804
Clinical protocol					< 0.01
Long agonist protocol	123 (28.8%)	499 (57.8%)	548 (71.3%)	271 (72.5%)	
Short agonist protocol	63 (14.8%)	163 (18.9%)	105 (13.7%)	52 (13.9%)	
Prolonged agonist protocol	6 (1.4%)	12 (1.4%)	13 (1.7%)	9 (2.4%)	
Other	235 (55.0%)	190 (22.0%)	103 (13.4%)	42 (11.2%)	
Embryo transferred	0.53 ± 0.81	0.87 ± 0.92	1.05 ± 0.93	1.07 ± 0.92	< 0.01
Progesterone(ng/ml)	1.49 ± 2.87	1.51 ± 3.05	1.24 ± 1.29	1.18 ± 1.60	0.0352
Clinical pregnancy	32 (7.5%)	161 (18.6%)	214 (27.8%)	132 (35.2%)	< 0.01

^a^One-way ANOVA. Values are mean + SD

^b^Pearson χ^2^ test. Values are number (percentage)

For patients with a hypoallergenic pattern (N = 2002) (Table 7), there was a negative association between age and EMT (32.4 vs. 31.9 vs. 30.9 vs. 30.7; *P* < 0.001). Unlike other endometrial patterns, these patients had similar infertility durations (*P* > 0.05). Clinical pregnancy rates were still significantly higher in patients with thicker EMT (2.6% vs. 14.1% vs. 23.8% vs. 30.1%; *P* < 0.001).

**Table 7:** Predictors and clinical outcome according to EMT with hypoallergenic pattern

Variables	Group1	Group2	Group3	Group4	*P* value
	(N = 549)	(N = 631)	(N = 543)	(N = 279)	
Age (years)	37.77 ± 5.88	34.03 ± 6.44	31.6 ± 5.31	31.3 ± 5.00	< 0.01
Duration of infertility (years)	4.16 ± 4.17	4.16 ± 3.86	4.14 ± 3.46	4.21 ± 3.37	0.994
Clinical protocol					< 0.01
Long agonist protocol	49 (9.0%)	202 (32.0%)	249 (45.9%)	140 (50.2%)	
Short agonist protocol	47 (8.6%)	125 (19.8%)	136 (25.0%)	67 (24.0%)	
Prolonged agonist protocol	30 (5.5%)	60 (9.5%)	85 (15.7%)	45 (16.1%)	
Other	423 (77.0%)	244 (38.7%)	73 (13.4%)	27 (9.7%)	
Embryo transferred	0.16 ± 0.53	0.55 ± 0.85	0.84 ± 0.94	0.94 ± 0.95	< 0.01
Progesterone (ng/ml)	1.11 ± 1.21	1.36 ± 1.91	1.41 ± 2.12	1.29 ± 2.61	0.0418
Clinical pregnancy	14 (2.6%)	89(14.1%)	129 (23.8%)	84 (30.1%)	< 0.01

^a^One-way ANOVA. Values are mean + SD

^b^Pearson χ^2^ test. Value are number (percentage)

Tables 8, 9 and 10 show the basic indicators and pregnancy results for different EMT groups. The indicators include endometrial pattern, age, duration of infertility, number of embryos transferred, progesterone levels, and pregnancy outcomes for each clinical protocol (long agonist protocol, short agonist protocol, and prolonged agonist protocol).

**Table 8:** Predictors and clinical outcome according to EMT with Long agonist protocol

Variables	Group1	Group2	Group3	Group4	*P* value
	(N = 396)	(N = 2578)	(N = 2770)	(N = 981)	
Age (years)	30.89 ± 5.88	30.22 ± 5.02	29.89 ± 4.44	29.89 ± 4.56	< 0.01
Duration of infertility (years)	3.49 ± 3.98	3.91 ± 3.16	3.84 ± 2.95	4.01 ± 2.55	0.00188
Endometrial patterns					< 0.01
A	224 (56.6%)	1877 (72.8%)	1973 (71.2%)	570 (58.1%)	
B	123 (31%)	409 (15.9%)	548 (19.9%)	271 (27.6%)	
C	49 (12.4%)	202 (7.8%)	249 (9%)	140 (14.3%)	
Embryo transferred	1.06 ± 0.85	1.19 ± 0.88	1.21 ± 0.91	1.17 ± 0.88	< 0.01
Progesterone(ng/ml)	1.09 ± 2.22	1.1 ± 0.44	1.05 ± 0.42	1.03 ± 0.38	0.181
Clinical pregnancy	74 (18.7%)	831 (32.2%)	1006 (36.3%)	391 (39.9%)	< 0.01

**Table 9:** Predictors and clinical outcome according to EMT with short agonist protocol

Variables	Group1	Group2	Group3	Group4	*P* value
	(N = 293)	(N = 1082)	(N = 830)	(N = 240)	
Age (years)	35.65 ± 5.43	33.39 ± 5.72	32.55 ± 5.65	33.27 ± 5.52	< 0.01
Duration of infertility (years)	4.29 ± 1.26	4.31 ± 3.81	4.61 ± 3.82	4.23 ± 3.26	0.29
Endometrial patterns					< 0.01
A	171 (58.4%)	806 (74.5%)	589 (71.0%)	121 (50.42%)	
B	72 (24.6%)	154 (14.2%)	105 (12.7%)	52 (21.7%)	
C	50 (17.1%)	122 (11.3%)	136 (16.4%)	67 (27.9%)	
Embryo transferred	0.87 ± 0.93	1.05 ± 0.92	1.08 ± 0.92	1.05 ± 0.92	< 0.01
Progesterone(ng/ml)	0.96 ± 0.51	1.04 ± 0.57	1.04 ± 0.53	1.00 ± 0.47	0.13
Clinical pregnancy	36 (12.3)	238 (22.0%)	228 (27.5%)	73 (30.4%)	< 0.01

**Table 10:** Predictors and clinical outcome according to EMT with prolonged agonist protocol

Variables	Group1	Group2	Group3	Group4	*P* value
	(N = 100)	(N = 424)	(N = 646)	(N = 252)	
Age (years)	31.81 ± 5.93	30.62 ± 4.83	29.8 ± 4.34	29.76 ± 4.39	< 0.01
Duration of infertility (years)	3.49 ± 3.71	3.51 ± 3.24	3.72 ± 2.77	3.42 ± 2.55	0.00103
Endometrial patterns					< 0.01
A	64 (64%)	352 (83%)	548 (84.8%)	198 (78.62%)	
B	6 (6%)	12 (2.8%)	13 (2%)	9 (3.6%)	
C	30 (30%)	60 (14.2%)	85 (13.2%)	45 (17.9%)	
Embryo transferred	0.91 ± 0.72	1.13 ± 0.83	1.16 ± 0.78	1.17 ± 0.91	< 0.01
Progesterone(ng/ml)	1.23 ± 1.99	1.14 ± 0.44	1.11 ± 0.42	1.07 ± 0.36	0.196
Clinical pregnancy	25 (25%)	166 (39.2%)	270 (41.8%)	104 (41.2%)	< 0.01

### Influence of different factors on pregnancy outcomes

Seven indicators were included in the predictive model: age, duration of infertility, EMT, endometrial pattern, number of large follicles, treatment plan, and number of embryos transferred. The optimal regression equation is:

$\hat{Y}$ = 21.587 + 0.063* (for 25–30 year old group) + 0.034* (for 30–35 year old group) − 0.183* (for 35–40 year old group) − 1.375* (for ≥ 40 year old group) − 0.081* (for < 1 year) − 0.104* (for 1–3 years) − 0.242* (for 3–6 years) − 0.422* (for 6–9 years) − 0.606* (for ≥ 9 years) + 0.610* (intima thickness is 2) + 0.806* (intima thickness is 3) + 1.105* (intima thickness is 4) − 0.229* (endometrial pattern B) − 0.135 * (endometrial pattern C) + 0.219 * (5–9) + 0.326* (10–14) + 0.271* (≥ 15) -0.232* (short program) + 0.411* (long program) + 20.154* (embryo transfer is 1) + 21.041* (embryo transfer is 2) + 20.499* (embryo transfer is 3).

The probability of a positive result (successful pregnancy):

$$P = \frac{1}{{1 + \exp \left( { - \hat{Y}} \right)}}$$

The likelihood ratio test statistic of this model is 9395.554, the degree of freedom is 24, *P* < 0.001, and the regression equation is highly significant. The Hosmer–Lemeshow goodness-of-fit test statistic of this model is 6.455, and the *P* value is equal to 0.596, which indicates that there is no statistically significant difference between the expected frequency and the observed frequency obtained by the prediction probability. In other words, the model fits well.

**Fig. 3:**
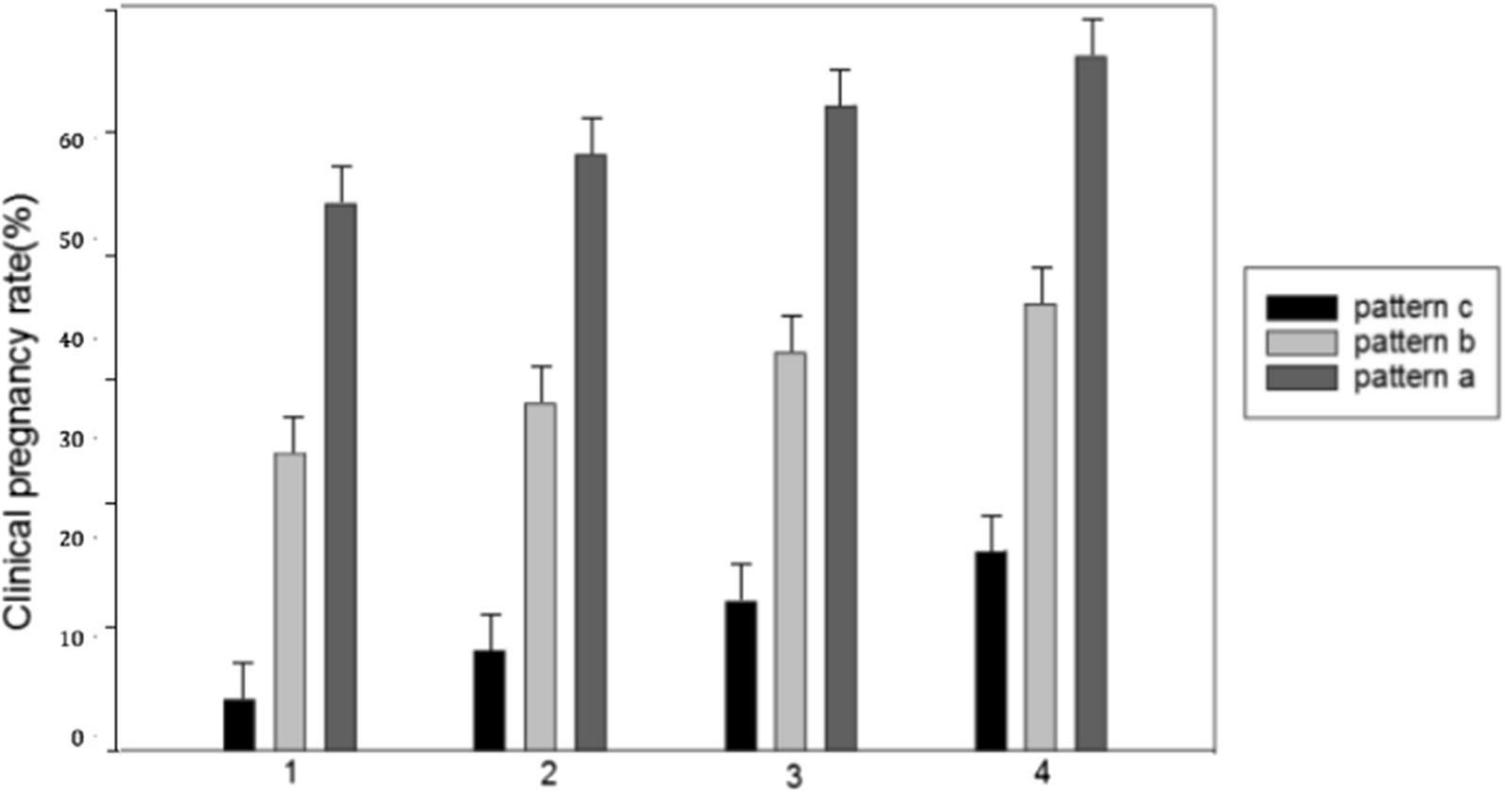
Distribution of pregnancy rates of three endometrium types in four groups of patients

We compared the effects of three different endometrial patterns on pregnancy outcomes (Fig. 3). Significant differences were reported in the clinical pregnancy rate, as follows:

- Pattern A: group 1 (12.2%), group 2 (30.8%), group 3 (37.4%), and group 4 (40.5%);
- Pattern B: group 1 (7.5%), group 2 (18.6%), group 3 (27.8%), and group 4 (35.2%); and
- Pattern C: group 1 (2.6%), group 2 (14.1%), group 3 (23.8%), and group 4 (30.1%).

Multivariate logistic regression analysis (Table 11) indicates that cycle protocols (short agonist protocol: OR 0.7; 95% CI 0.62–0.8), EMT (OR 1.12; 95% CI 1.09–1.14), age (OR 0.96; 95% CI 0.94–0.97), duration of infertility (OR 0.97; 95% CI 0.95–0.98), progesterone levels (OR 0.63; 95% CI 0.54–0.73), number of embryos transferred (OR 7.12; 95% CI 6.52–7.77), and endometrial pattern (pattern B: OR 0.8; 95% CI 0.7–0.92) were predictive of pregnancy outcomes.

**Table 11:** Logistic Regression Analysis for the association between potential predictors and clinical pregnancy

Predictors	OR (95%CI)	*P*
Clinical protocol		< 0.001a
Long agonist protocol	*	
Short agonist protocol	0.7 (0.62, 0.8)	< 0.001
Prolonged agonist protocol	1.4 (1.2, 1.64)	< 0.001
Other	0.85 (0.7, 1.03)	0.1
Duration of infertility	0.97 (0.95, 0.98)	< 0.001
Endometrial thickness	1.12 (1.09, 1.14)	< 0.001a
Endometrial morphology		< 0.001
a	*	
b	0.8 (0.7, 0.92)	0.001
c	0.72 (0.61, 0.86)	< 0.001
Age	0.96 (0.94, 0.97)	< 0.001
Progesterone	0.63 (0.54, 0.73)	< 0.001
ET	7.12 (6.52, 7.77)	< 0.001

^*^Reference group

^a^P-value of each variables overall effects after adjusting for the other variables

^b^P-value between each variable subgroups and reference group

"ET" means number of embryos transferred

**Fig. 4:**
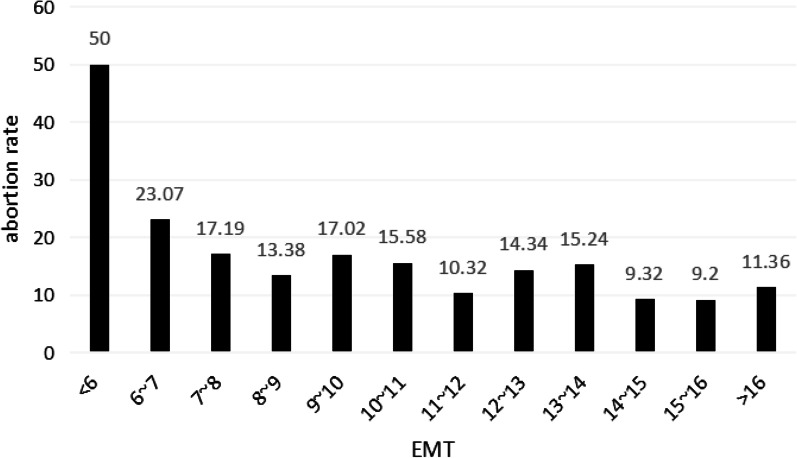
Predictions of miscarriage rate at different EMT thresholds

Previous research has mentioned a relationship between abortion and EMT, so we analyzed this too (Fig. 4). When the EMT was less than 6 mm, the miscarriage rate was as high as 50%, and tended to be stable as the EMT increased (*P* < 0.05).

### Prediction results

We calculated the probability of pregnancy in each treatment cycle before the patient entered treatment. We sorted each predicted value from smallest to largest, then used each decile as a cutoff to sort the predicted probabilities into groups. We used the nine decile points to obtain 0.0000, 0.0000, 0.0000, 0.0000, 0.2845, 0.4218, 0.5227, 0.5742, and 0.6255 and divided the predicted probability values into 10 groups. As the sample size was too large, we found the first four *P* values were close to zero after we sorted them from smallest to largest. We combined them into one group for a total of six groups.

**Fig. 5:**
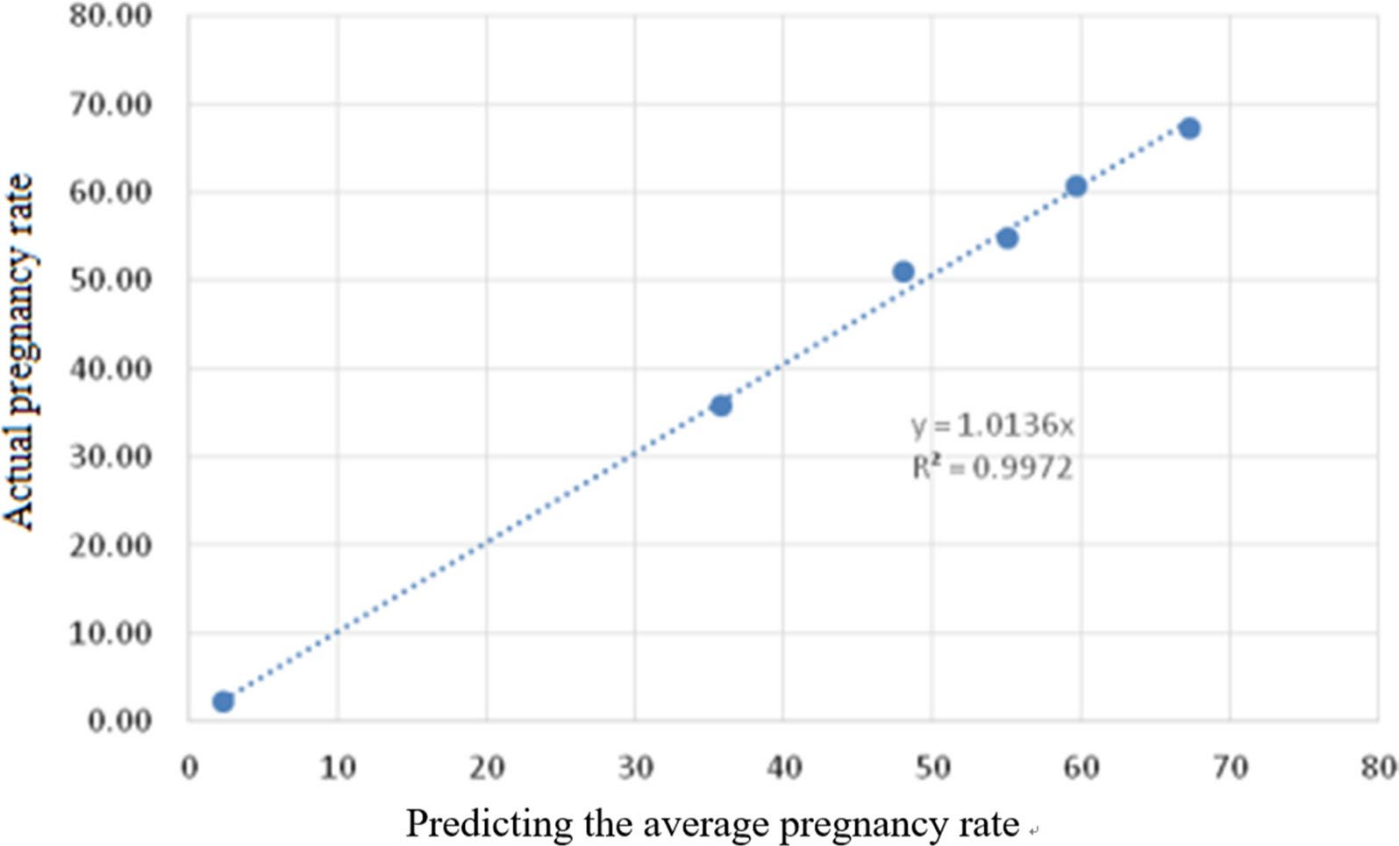
Estimation of the average pregnancy rate and the actual observed pregnancy rate scatter plot. The correlation equation shows the statistic relationship between the average value of the predicted pregnancy rate and the actual pregnancy rate of each group, and the correlation coefficient is calculated to obtain R^2^ = 1, *P* < 0.001, indicating that there is a correlation between the predicted pregnancy rate averages and the correlation

The actual pregnancy rate can be obtained by calculating the number of actual pregnancies in the group and the ratio of the total number of cases in each group. The average predicted pregnancy rate and the average predicted number of pregnancies were calculated in each group. The actual number of pregnancies per group (average predicted number of pregnancies) can be used to obtain the error rate for each group of predictions (Table 12). The prediction error rate of each group was within ± 4%, and the maximum error was 3.02% for the third group. The overall prediction error rate is 0.41. The average prediction error of each group is taken as an absolute value, 0.72%. In the coordinate system, the average predicted pregnancy rate is used as the axis coordinate point, and the actual observed pregnancy rate value is plotted as the axis coordinate point. The correlation equation is obtained by bivariate correlation using the average predicted pregnancy rate and the actual pregnancy rate of each group (Fig. 5). The correlation coefficient was calculated to obtain R^2^ = 1, *P* < 0.001, indicating that there is a close correlation between the predicted pregnancy rate averages.

**Table 12:** Predicted pregnancy rate actual pregnancy rate between groups

Group	Predicted pregnancy rate (%)	Average predicted pregnancy rate (%)	Average predicted number of infants	Actual pregnancy	The total number of cycles in the group	Actual pregnancy rate (%)	Error rate (%)
1	< 28.45	2.26	147	151	6499	2.32	− 0.06
2	28.45–42.18	35.81	466	466	1301	35.81	0
3	42.18–52.27	48.07	614	653	1278	51.09	− 3.02
4	52.27–57.42	55.06	730	728	1325	54.94	0.01
5	57.42–62.55	59.67	766	781	1284	60.82	− 1.15
6	> 62.55	67.35	879	877	1304	67.25	0.10
Total	< 62.55	27.73	3602	3656	12,991	28.14	− 0.41

## Discussion

### Main findings

In this retrospective study, we listed many possible predictors of pregnancy outcome for 12,991 patients undergoing IVF-ISCI. Our study demonstrated that EMT and clinical pregnancy rates are positively correlated. The logistic regression analysis showed that, within all of the other relative IVF factors, EMT was an independent and vital predictor of pregnancy outcomes.

In our study, we used 8 mm as the cut-off value for a thin endometrium. The pregnancy rate increased rapidly when EMT reached 8 mm (pattern A: 12.2% vs. 30.8%; pattern B: 7.5% vs. 18.6%; pattern C: 2.6% vs. 14.1%). Patients with thinner EMT were significantly older. Pattern C on the trigger day suggested a significantly low clinical pregnancy rate. We found a consistent positive correlation between EMT and clinical pregnancy rate in patients with pattern B or pattern C. When the EMT was below 14 mm, the pregnancy rate ranking among the different endometrial patterns was pattern A > pattern B > pattern C. When EMT reached 14 mm, clinical pregnancy rates were similar in the three different endometrial patterns.

For patients with different endometrial patterns, the factors of age, duration of infertility, clinical protocol choice, progesterone dose, and the number of embryos transferred were compared between the four groups. Only the clinical protocol choice indicated significant differences in every type of endometrium. Patients with prolonged agonist protocol had a rather low clinical pregnancy rate. The clinical pregnancy rate did not improve with the increase in EMT. However, most patients who chose the long agonist protocol had better clinical pregnancy rates. Therefore, despite other possible factors, the long agonist protocol was recommended in most situations.

### Strengths and limitations

Our retrospective study is more comprehensive than previous research. We included patients with poor physical condition (a BMI range from 14.7 to 43, age range from 20 to 51) for more convincing results. Moreover, few studies have compared pregnancy rate and EMT in three different endometrial patterns. However, the relationship between a thick endometrium and clinical pregnancy rate is controversial. We found that the two vital predictors should be discussed separately. Most previous studies only considered treatment with a standard GnRH-a long protocol. In our study, we examined the long agonist protocol, short agonist protocol, and prolonged agonist protocol.

Our study had some limitations. All indicators, such as E2, progesterone, EMT, and endometrial patterns, were measured on the same day. The predictive values at other time points (baseline, day three of stimulation, etc.) should be examined.

## Conclusion

We established and evaluated a predictive model of the factors influencing pregnancy rate. The predictive model has excellent discriminative ability regarding the patient's final pregnancy outcome. The pregnancy probability of each cycle calculated by the predictive model roughly reflects the true pregnancy probability of the patient. The smaller the prediction error, the more accurate the prediction result. Therefore, the established model can be used to assess patient prognosis before certain treatments. Patients can be classified as having a low to high pregnancy rate. This model provides a means of communication and decision-making for doctors and patients.

Our examination of the relationship between EMT and IVF outcomes revealed that different endometrial patterns exhibited different characteristics. Factors such as age, cycle protocols, duration of infertility, progesterone levels, and the number of embryos transferred played an important role in predicting pregnancy outcomes. When the EMT is less than 6 mm, we should remind the patient that their miscarriage rate is high. Thick EMT positively affected clinical pregnancy rates, which did not drop when EMT reached 14 mm. This was consistent with some studies that demonstrated no reduction in pregnancy rate in cases of a very thick endometrium [18].

## Data Availability

The data generated or analyzed during this study is available from the corresponding author upon reasonable request.

## Abbreviations

EMT: endometrial thickness
IVF: in vitro fertilization
HCG: human chorionic gonadotropin
ART: assisted reproductive technology
TVU: all transvaginal ultrasound
Gn: gonadotropin
