# Correction: Analysis of endometrial thickness patterns and pregnancy outcomes considering 12,991 fresh IVF cycles

**DOI:** 10.1186/s12911-025-03185-3

**Published:** 2025-09-24

**Authors:** ShuJie Liao, Renjie Wang, Cheng Hu, Wulin Pan, Wei Pan, Dongyang Yu, Lei Jin

**Affiliations:** 1 Department of Obstetrics and Gynecology, Tongji Hospital, Tongji Medical College, Huazhong University of Science and Technology, Wuhan, 430030 Hubei People’s Republic of China; 2 School of Economic and Management, Wuhan University, Wuhan, 430072 People’s Republic of China; 3 School of Applied Economics, Renmin University of China, Beijing, 100872 People’s Republic of China

**Correction to: Liao et al. BMC Med Inform Decis Mak (2021) 21:176**

10.1186/s12911-021-01538-2

Following the publication of the original article, the author reported that Table 8 was erroneously duplicated as Table 9. The correct Table 9 is as follows.

**Table 9:** Predictors and clinical outcome according to EMT with short agonist protocol

Variables	Group1 (*N* = 293)	Group2 (*N* = 1082)	Group3 (*N* = 830)	Group4 (*N* = 240)	*p* value
Age (years)	35.65 ± 5.43	33.39 ± 5.72	32.55 ± 5.65	33.27 ± 5.52	< 0.01
Duration of infertility (years)	4.29 ± 1.26	4.31 ± 3.81	4.61 ± 3.82	4.23 ± 3.26	0.29
Endometrial patterns					< 0.01
A	171(58.4%)	806(74.5%)	589(71.0%)	121(50.42%)	
B	72(24.6%)	154(14.2%)	105(12.7%)	52(21.7%)	
C	50(17.1%)	122(11.3%)	136(16.4%)	67(27.9%)	
Embryo transferred	0.87 ± 0.93	1.05 ± 0.92	1.08 ± 0.92	1.05 ± 0.92	0.01
Progesterone (ng/ml)	0.96 ± 0.51	1.04 ± 0.57	1.04 ± 0.53	1.00 ± 0.47	0.13
Clinical pregnancy	36(12.3)	238(22.0%)	228(27.5%)	73(30.4%)	< 0.01

The original article has been updated accordingly.

